# Measuring anxiety related to COVID-19: Factor analysis and psychometric properties of the Arabic Coronavirus Anxiety Scale

**DOI:** 10.1371/journal.pone.0260355

**Published:** 2021-11-24

**Authors:** Zahir Vally, Aisha Alowais

**Affiliations:** 1 Faculty of Medicine and Health Sciences, Department of Clinical Psychology, United Arab Emirates University, Al Ain, UAE; 2 Wolfson College, University of Oxford, Oxford, United Kingdom; St John’s University, UNITED KINGDOM

## Abstract

Literature reports that fear and anxiety related to the coronavirus (COVID-19) pandemic may be a significant factor in promoting adherence to health-protective behaviours. This study aimed to validate an Arabic version of the Coronavirus Anxiety Scale (CAS). Participants aged 18 to 58 years of age were recruited from a university population (students and staff) as well as via social media from 22 June to 18 July 2020 when the United Arab Emirates was under a partial government-instituted lockdown. They completed Arabic versions of the CAS and the Fear of COVID-19 Scale. A confirmatory factor analysis produced a unidimensional structure and all items satisfactorily loaded onto this single factor (i.e., the physiological symptoms of fear and anxiety related to coronavirus). The Arabic CAS was internally consistent and concurrently valid. These preliminary findings suggest that the Arabic CAS is a valid and reliable instrument to employ in the assessment of dysfunctional anxiety related to coronavirus. The availability of this validated measure will enable the further conduct of a variety of mental health studies in relation to the COVID-19 pandemic. It also holds clinical utility as a potential screening measure for those afflicted by anxiety symptomology during the pandemic.

## Introduction

On the 11^th^ March 2020, the World Health Organization (WHO) [1] announced that the novel coronavirus (COVID-19), which had originated in Wuhan (China) in December 2019, was considered a pandemic. At the time of the announcement, the virus had rapidly spread to most parts of the globe, with the most severe rates of prevalence observed in North America and Europe. At the time of writing this manuscript, 5 months following the WHO’s announcement, more than 21 million global cases have been reported, 771 114 individuals have died, with the United States, Brazil and India having been most severely impacted by the pandemic in terms of mortality [2].

The United Arab Emirates (UAE), the location of the present study, has also been impacted by the pandemic. On August 19^th^ 2020 (at the time of writing), 65 341 positive cases had been diagnosed and 367 deaths had been recorded [2]. The UAE government instituted a number of measures within the first few days following the WHO’s announcement in March 2020 in an attempt to curtail the spread of the disease. All schools and educational institutions were closed and moved to virtual delivery of classes and lectures. On March 15^th^, public sector institutions and retailers reduced their hours of operation and remote working arrangements were implemented where feasible. On March 25^th^, all shopping malls, commercial centres, places of worship, leisure centres, and all retailers, with the exception of supermarkets and pharmacies, were closed. Dubai and Abu Dhabi airports ceased all flights and were both closed. A nationwide program of disinfection commenced during which time a mandatory night-time curfew was implemented, violation of which carried substantial fines.

The fear created by the COVID-19 pandemic and the commensurate impact of lockdown measures have had a multitude of consequences for the mental health and well-being of entire societies, impacting communities at every possible level [3]. Those with vulnerabilities created by pre-existing psychological and physical health conditions, individuals with inadequate social support networks, and those deployed to work on the frontlines during this pandemic (e.g., first responders, healthcare workers) are at particularly high risk for developing mental health difficulties [4,5]. There have been some reports, from varying locations across the world, of individuals committing suicide who feared that they had contracted COVID-19 but, following an autopsy, this was not found to be the case [6]. At the height of the pandemic, most countries introduced lockdown measures and remote working arrangements for large proportions of society, the amount of time spent at home rose dramatically, resulting in an absence of normalcy, structure, routine, and purpose. For some individuals, harmful and potentially fatal consequences ensued. For example, the rate of domestic violence appears to have increased during the lockdown period [7]. The mandated isolation that resulted from the lockdown and the sometimes-enforced constant contact with others (in the form of stay-at-home orders) may also have resulted in frustration and social conflict. Moreover, the prevailing sense of uncertainty in relation to the future that now still persists and, for many, the financial insecurity that has resulted from the economic implications of the pandemic as many individuals have become unemployed, may also be catalysts for spiralling stress, escalating anxiety, and a preponderance of depressive feelings [8–10]. To this end, the WHO established a set of guidelines for the period of home confinement consisting of recommendations directed at the promotion of physical and mental health and towards managing the task of parenting during the atypical circumstances of the pandemic [11].

Preliminary evidence appears to support the contention that the pandemic has precipitated mental health difficulties for many individuals and this evidence pervades across a great many countries and regions of the world. For example, in China, substantial rates of traumatic stress (73.4%), depression (50.7%), generalized anxiety (44.7%), and insomnia (36.1%) have been reported amongst medical personnel [12]. A recent study in the United States reported that individuals who scored highly on a measure of coronavirus anxiety also demonstrated elevated levels of despair, suicidal ideation, religious crisis, and the use of alcohol and/or substances as a means of coping [13]. These psychological reactions and the extent thereof are similar to those that were observed during previous large-scale outbreaks of disease such as during the Severe Acute Respiratory Syndrome outbreak [14]. Some researchers have suggested that the presence of a moderate degree of anxiety may in fact be functional within the context of an outbreak of disease as it may promote a healthy degree of caution and compliance with virus-mitigating behaviours [5]. However, the presence of substantial levels of fear and anxiety may impede individuals’ capacity for sound decision-making and disrupt their mental health [3]. This may particularly be the case when paying excessive and obsessive attention to information related to COVID-19 [15]. Moreover, individuals’ personal experiences of the pandemic and the nature of their exposure to information, either via exposure in the media or through first-hand experiences, may also serve to further amplify their fear and anxiety [16–18]. For example, preliminary evidence demonstrates that being diagnosed with COVID-19, experiencing the symptoms oneself, or a loved one or acquaintance having had the diagnosis precipitates higher rates of coronavirus-related anxiety compared to individuals who have not been exposed to these experiences [19].

Despite the clear need for psychological intervention for those impacted by the pandemic, Xiang et al. [20] suggests that the mental health needs of individuals, particularly during the initial stages of the pandemic, were notably neglected. The scope of the pandemic and its associated rate of infection and mortality meant that the explicit focus of healthcare providers and researchers was on infection control and the development of a vaccine. However, mental healthcare professionals are now becoming increasingly aware of the mental health burden that the pandemic has precipitated, and as such, an expanding selection of assessment instruments has steadily proliferated specifically designed to assess mental health symptoms related to coronavirus [3,13,21,22] as well as the typical coping strategies individuals tend to employ in attempting to manage the deleterious mental health consequences associated with the pandemic [23,24]. One such measure, the Coronavirus Anxiety Scale (CAS), developed by Lee [13], has consistently been shown to be valid and highly reliable. The original validation study for the English CAS reports that the measure possesses a stable unidimensional and invariant factor structure (across gender, race, and age) and the diagnostic accuracy of other analogous psychiatric screening instruments. Moreover, it is reliable (α = .92), and its construct validity has been demonstrated with positive associations across a wide range of variables indicative of functional impairment and psychological distress, specifically, depression, generalized anxiety, anxiety related to death and health, a previous history of anxiety difficulties, a positive coronavirus diagnosis, COVID-19 related fear, a sense of hopelessness, and suicidal ideation. These associations were consistently present over and above any sociodemographic, vulnerability, or COVID-19 related factors. It is also capable of identifying functional impairment with a reliable cut-off score (76% sensitivity and 90% specificity) [25,26]. The development of this measure is an essential contribution to the assessment of anxiety related to COVID-19 which will directly inform the clinical practice of clinicians working with individuals impacted by their experience of the pandemic. However, while the original English instrument has been validated in additional languages (e.g., Turkish [19] and Bangla [27]), no Arabic version has yet been subjected to systematic validation. The residents of 25 countries in the Arabian Peninsula, North Africa and the Middle East speak Arabic as either an official or co-official language, an approximate 422 million people [28]. This is a substantial proportion of people who may potentially benefit from the availability of a psychometrically valid instrument to assess COVID-related anxiety.

Therefore, the principal aim of the present study was to translate the CAS measure to Arabic and investigate its psychometric properties. To this end, we proposed the following hypotheses: Following a factor analysis, the Arabic CAS will produce a unidimensional structure similar to its previous language versions with acceptable factor loadings and will meet multiple indices of acceptable model fit (Hypothesis 1), the measure will be internally consistent with *α* ≥ 0.7 (Hypothesis 2), and it will be concurrently valid when correlated with a second instrument that measures a similar and related psychological construct (Hypothesis 3).

## Methods

### Procedure and participants

The study’s protocol received ethical approval for its conduct from the Social Sciences Research Ethics Sub-committee at the author’s institution (Reference Number: ERS_2020_6102). The study conformed to the stipulations of the 1964 Declaration of Helsinki and its later amendments. Data were collected from Arabic-speaking participants resident in the United Arab Emirates during an approximately 4-week period of time (22 June to 18 July 2020). Given the rapidly evolving nature of the pandemic and the steady proliferation of research being conducted on the subject, including in the Middle East, we felt it was prudent to complete data collection as rapidly as possible to ensure that the translated measure could become available to researchers and clinicians as soon as possible. Participants were approached via a combination of convenience sampling (university staff and students at the authors’ institution) and via posts placed on social media. For the latter strategy, the authors wrote to the administrators of popular health and wellness accounts requesting their permission to post an advertisement about the study. Participants provided informed consent, were duly informed of their rights as participants in the study and the responsibilities of the research team and were provided with the contact details of the principal investigator.

A cross-sectional online survey was administered to assess the factor structure and psychometric properties of an Arabic-translated version of the CAS. A total of 237 participants completed the questionnaire. The sample ranged in age from 18 to 58 years of age (*M*_age_ = 29.47, SD = 9.34). The majority of the sample were females (*n* = 145, 61.2%). We assessed level of education by querying the number of years of formal education, for example, an individual who had completed high school but had not yet completed the first year of university was recorded as having completed 12 years of education. This procedure resulted in a continuous variable for which years of education for this sample ranged from 12 to 21 years (*M* = 15.42, SD = 1.78). In terms of employment status, 96 participants (40.5%) were employed fulltime, 13.5% (*n* = 32) were employed part-time, a further 40.5% (*n* = 96) were presently fulltime students, and the remaining 5.5% (*n* = 13) were unemployed at the time of data collection. Eighteen participants (7.6%) reported that they were at present or had previously received a positive COVID-19 diagnosis and 21.1% of the sample (*n* = 50) reported having a friend, family member, or acquaintance who was at present or had previously tested positive for COVID-19. Finally, 40 participants (16.9%) reported having previously experienced issues with anxiety difficulties for which they had sought professional treatment. The sample’s demographic characteristics are summarized in Table 1.

**Table 1:** Socio-demographic variables, total sample and stratified by gender.

	Total (n = 237)	Males (n = 92)	Females (n = 145)
Age in years (Mean ± SD)	29.47 (9.34)	30.54 (9.20)	28.79 (9.40)
Years of formal education (Mean ± SD)	15.42 (1.78)	15.89 (1.67)	15.12 (1.79)
Employment status			
Working fulltime	96 (40.5)	56 (60.9)	40 (27.6)
Working part-time	32 (13.5)	9 (9.8)	23 (15.9)
Fulltime student	96 (40.5)	27 (29.3)	69 (47.6)
Unemployed	13 (5.5)	0 (0.0)	13 (9.0)
Positive COVID-19 diagnosis	18 (7.6)	7 (7.6)	11 (7.6)
Relative or acquaintance with COVID-19 diagnosis	50 (21.1)	20 (21.7)	30 (20.7)
History of an anxiety disorder	40 (16.9)	3 (3.3)	37 (25.5)

*Note*. Data are mean and standard deviation for continuous variables and count and percentage for categorical variables.

### Assessment instruments

#### Demographic information

Participants provided responses to prompts for the following demographic information: age, sex, duration of education (number of years), current employment status, the presence of a COVID-19 diagnosis, history of an anxiety diagnosis, and if they had a relative or acquaintance with a COVID-19 diagnosis.

#### The Coronavirus Anxiety Scale

To assess coronavirus-related anxiety, we translated the original English version of the Coronavirus Anxiety Scale (CAS) [13,25,26], which was developed following the assessment of adults resident in the United States, and then by factor analyzing an item pool reflecting various anxiety symptoms. The most recent English version of the CAS employs a revision to the wording of the instructions, and the items now refer to a 1-week time frame for the assessment of symptoms, rather than the original 2-week period. This change was exacted in response to the evolving nature of the pandemic in which individuals’ experience of the stressors related to the pandemic and their magnitude appear to change as the pandemic progresses. The CAS consists of 5 items accompanied by a 5-point Likert scale (0  =  *Never* to 4  =  *Every day*) that measures the presence and frequency of anxiety symptoms precipitated by attending to the pandemic (operationalized as thinking, reading, listening, or consuming information about COVID-19). The 5 items capture 5 principal symptoms of coronavirus-related anxiety: specifically, difficulty sleeping, feeling paralyzed/frozen, nausea/stomach problems, loss of appetite, and dizziness/light-headedness/faintness. Scores to the items are tallied with higher scores indicative of greater coronavirus-related anxiety. A cut-off score of 9 or higher on the CAS has been shown to indicate clinically dysfunctional levels of coronavirus-related anxiety, and for respondents who evidence scores within this clinical range, significant associations with suicidal ideation, drug/alcohol use, depression, and generalized anxiety are also evident [13,25,26,28].

We approached the author of the original CAS measure to elicit his permission to translate and validate an Arabic version. The following procedure was followed to arrive at an acceptable translated version of the CAS. The English items were translated to Arabic using the back-translation method outlined by Brislin [29]. First, a bilingual (Arabic and English) translation expert, an individual with a graduate degree in translation studies, translated the items from English to Arabic resulting in a forward-translated version. A second individual, with the same credentials, back translated the items, from Arabic to English. Both versions, the forward and back-translated documents, were compared for equivalence, the cultural appropriateness of the items was assessed, and any discrepancies between the two versions were reviewed and edited, resulting in a final accepted Arabic version based on consensus (Appendix 1 in S2 File).

#### The Fear of COVID-19 Scale

The Arabic version of the Fear of COVID-19 Scale (FCS) [30] was administered as this would serve as a measure from which the concurrent validity of the translated CAS could be determined. The FCS is a 7-item scale that is scored using a 5-point Likert scale that ranges from 1 (strongly disagree) to 5 (strongly agree). Scores can range from 7 to 35 with higher scores indicative of greater fear related to COVID-19. The scale has been translated to a number of languages and consistently displays a unidimensional structure and robust psychometric properties across all language versions [3]. For the Arabic version, corrected item-total correlations ranged from .57 to .74, factor loadings for each item ranged from .62 to .84, and internal consistency was high (*α* = .88). The measure’s concurrent validity has been demonstrated by significant correlations with indicators of depression, anxiety, and psychological distress [30]. In the present study, internal consistency was similarly excellent (*α* = .91).

### Data analytic strategy

Descriptive statistics are reported using counts and percentages for categorical variables and means and standard deviations for continuous variables. The data distributions for each of the two primary outcome variables, the CAS and the FCS, and for each of the five CAS items were examined for normality by computing skewness and kurtosis values. None of the skewness or kurtosis values exceeded 2.0, indicating that the data did not deviate from normal [31]. To determine whether the newly translated CAS possessed structural validity, a confirmatory factor analysis (CFA) was performed on the CAS data. Model fit was assessed using the maximum likelihood estimation and a number of additional indices with recommendations for the minimum criteria for acceptable model fit [32–34]. Internal consistency for each measure is reported using Cronbach’s alpha coefficients (*α*), inter-item correlations and corrected item-total correlations. The convergent validity of Arabic CAS was assessed by computing a Pearson’s *r* value between the CAS and the Arabic FCS. Fornell and Larcker’s [35] average variance extracted value and a composite reliability value were also computed. Differences in the total CAS score were examined in relation to the demographic variables (i.e., gender, job status, COVID-19 diagnosis, friend/acquaintance with COVID-19 diagnosis, pre-existing anxiety disorder diagnosis) by computing an omnibus analysis of variance (ANOVA) in which all the examined demographic variables were included as independent variables. This was followed by post hoc tests to determine which of the included independent variables were statistically significant. For age and level of education which were measured as continuous variables, bivariate correlations were computed. All descriptive and psychometric analyses were conducted using the Statistical Package for the Social Sciences Version 26 [36] while the CFA was conducted using LISREL Version 10.20 [37].

## Results

### Factorial validity

We sought to confirm the unidimensionality of the Arabic CAS by conducting a CFA with maximum likelihood. In order to examine the extent to which the data fit the model estimated in the CFA, several indices of fit were examined and the following thresholds adopted to interpret the resulting output: a non-significant *χ*^*2*^ value (*p* > .05), *χ*^*2*^/df ≤ 3, Goodness of Fit Index (GFI), Adjusted Goodness of Fit Index (AGFI), Tucker-Lewis Fit Index (TLI) and Comparative Fit Index (CFI) > .90, and Root Mean Square Error of Approximation (RMSEA) and standardized root mean square residual (SRMR) values < .08 [32–34]. The estimation of the overall unidimensional model produced poor model fit (*χ*^*2*^ was significant [*p* < .05]; *χ*^*2*^/df = 30.36; GFI = .799; AGFI = .397; CFI = .853; TLI = .705; RMSEA = .352; SRMR = .056). The initial output suggested that, if the following modifications were made to the estimated model, the *χ*^*2*^ value could be decreased–we then set error covariances between items 2 and 3, between items 4 and 5, and between items 3 and 4. The recomputed model produced excellent model fit (*χ*^*2*^ was not significant [*p* = .35]; *χ*^*2*^/df = 1.055; GFI = .996; AGFI = .974; CFI = .990; TLI = .999; and RMSEA = .015; SRMR = .006). All the standardized factor loadings ranged from .78 to .86 and were therefore within the acceptable range [19]. These results are illustrated in Table 2.

**Table 2:** Confirmatory factor analysis fit indices of the Arabic Coronavirus Anxiety Scale.

Psychometric Test	Initial Model	Modified Model	Suggested cut-off score
*χ*^*2*^ (df)	151.80 (5)*	2.11 (2) *ns*	Nonsignificant
*χ*^*2*^*/*df	30.36	1.06	< 3.0
Comparative fit index	.853	0.990	> .90
Tucker-Lewis index	.705	0.999	> .90
Goodness of fit index	.799	0.996	> .90
Adjusted goodness of fit index	.397	0.974	> .90
Root-mean square error of approximation	.352	0.015	< .08
Standardized root mean square residual	.056	0.006	< .08

Note.

*p < 0.001; *ns* = nonsignificant; *χ*^*2*^ (df) = maximum likelihood ratio and degrees of freedom; CI = confidence interval.

### Internal consistency

The Cronbach’s alpha coefficient for the Arabic CAS was high (*α* = .89) indicating excellent reliability. Moreover, inter-item correlations for the 5 CAS items were all highly significant, the coefficient values ranged from .52 to .77 (*p* < .001). A reliability analysis also indicated that the overall internal consistency could not be significantly improved by deleting any single item from the scale (*α* values remained constant between .86 to .89) (see Table 3).

**Table 3:** Descriptive statistics, factor loadings, and reliability results of the Arabic Coronavirus Anxiety Scale.

						Inter-item Correlations			
CAS Item Description	M ± SD	Skewness/Kurtosis	Factor Loadings^a^	Item-Total Correlation	α if Item Deleted	2	3	4	5
**1. Dizzy/light-headed/faint**	1.05 ± .74	.88/1.59	.80	.647	.890	.60**	.56**	.56**	.52**
**2. Trouble sleeping**	1.22 ± 1.06	.50/-.80	.86	.790	.857	-	.77**	.63**	.63**
**3. Paralyzed/frozen**	1.87 ± 1.09	.05/-.85	.82	.769	.863		-	.64**	.60**
**4. Diminished appetite/eating**	1.19 ± .95	.77/.38	.78	.771	.861			-	.78**
**5. Nauseous/stomach problems**	1.28 ± .86	.65/.41	.78	.743	.869				-
**Total CAS**	6.61 ± 3.97								
**Cronbach’s alpha**	.89								

Note.

^a^Standardized factor loadings based on confirmatory factor analysis model 2

**All inter-item correlations were significant at *p* < .001; CAS = coronavirus anxiety scale; *α* = Cronbach’s alpha; CAS item descriptions have been truncated for brevity.

### Convergent validity

According to Fornell and Larcker [35], an instrument’s convergent validity can be determined by examining two variables, the average variance extracted (AVE) of the latent variable and the measure’s composite reliability (CR). Convergent validity can be considered adequate when the AVE value is ≥ .5 and the CR value is ≥ .7 [35]. The computed AVE value was well above the desired threshold (.75), so too was the CR value (.92), both confirming that the translated CAS possessed excellent convergent validity.

The convergent validity of the Arabic CAS was also assessed by correlating the total score for this scale with that of another related scale, the Arabic FCS. The magnitude of the correlational value was large and highly significant (*r* = .80, *p* < .001) indicating a substantial association between these two instruments. The results of these statistical tests support the validity of the newly translated Arabic CAS.

### Mean differences analyses

An omnibus ANOVA using linear regression was computed to determine whether the total CAS score would vary in relation to a number of independent variables; specifically, gender, job status, the presence or history of a COVID-19 diagnosis, the presence or history of a COVID-19 diagnosis in a close relative or acquaintance, and a previous history of anxiety difficulties. The overall ANOVA was highly significant (F(6, 230) = 22.600, p < .001) suggesting that differences in means were evident for some of the included variables.

Post hoc tests using a Bonferroni correction were then computed to investigate which of the included independent variables significantly differed. The results of these analyses demonstrated that females displayed comparably higher coronavirus-related anxiety than males (*F*(17, 219) = 1.703, *p* < .05). CAS scores were substantially higher among those who had been diagnosed with COVID-19 than those who had not (*F*(17, 219) = 13.717, *p* < .001). This was also the case for participants who had a relative or acquaintance who had been diagnosed with COVID-19 (*F*(17, 219) = 3.191, *p* < .001). Participants who reported a history of anxiety evidenced significantly higher CAS scores compared to those with no such history and this difference was statistically significant (*F*(17, 219) = 5.492, *p* < .001). Coronavirus anxiety did not differ as a function of job status.

We were also interested in assessing whether participants obtained varying CAS scores as a function of their age or level of education. As we measured these two demographic variables as continuous variables, we computed bivariate correlations between them and the total CAS score. A significantly positive relationship emerged between age and coronavirus anxiety (*r* = .33, *p* < .001) (i.e., generally, older individuals scored higher on the CAS measure), but no such relationship was evident for level of education.

## Discussion

The purpose of this study was to validate and evaluate the psychometric properties of an Arabic version of the CAS, a brief mental health screening measure that can be used to identify individuals experiencing probable dysfunctional anxiety in relation to the coronavirus pandemic. To do so, we conducted factor analyses using maximum likelihood to investigate the structure of the translated measure, which verified the measure’s unidimensional structure. Internal consistency was high (*α* = .89). Standardized factor loadings across the five items were large (λ = .78 to .86). Inter-item correlations were also all highly significant (*p* < .001) and moderate to large in magnitude (*r* = .52 to .78). The translated measure was therefore shown to be highly reliable and thematically consistent, with all items appearing to measure similar constructs (i.e., the distressing physiological symptoms associated with anxiety related to coronavirus).

This study’s data largely concurs with previous findings relating to the psychometric properties of the other language versions of the CAS. Validation studies conducted in Brazil [38], South Korea [39], Bangladesh [27], Turkey [19], and Colombia [40] all uniformly report the presence of a unidimensional structure and the internal consistency of their translated instruments to be high (Cronbach’s α values have been reported to range from .83 to .87). While the presence of coronavirus-related anxiety appears to occur pervasively across countries and cultures, the magnitude thereof is not consistent. Overall mean scores for the CAS in these previous validation studies have varied and, in some cases, quite substantially from as low as .87 in Bangladesh [27] to 2.46 in Colombia [40]. The overall mean score generated in the Arabic-speaking UAE sample is therefore substantially higher than that of previous studies with the exception of the Turkish study [19] in which a comparable score was produced (m = 6.66). It appears that residents of the UAE may be evidencing substantially higher levels of anxiety than elsewhere in the world perhaps as a result of the immensely strict levels of government control instituted in this country. For example, while many of the government-mandated rules here have been similar to that of other countries (e.g., mask-wearing in public spaces, curfews, closure of non-essential public spaces, limits on the number of people allowed to gather), contravention of these rules in this locale, carry substantial fines and in some cases, movement has been entirely restricted. The normalcy of life has therefore been changed dramatically and substantially.

Also, despite the easing of restrictions in some parts of the world, one cannot assume that anxiety will commensurately diminish, and individuals will no longer worry about the pandemic. Indeed, Nikčević and Spada [24] have identified a constellation of psychopathological symptoms and behaviours which they refer to as ‘COVID-19 anxiety syndrome’. They propose that this construct consists of a variety of maladaptive forms of coping with coronavirus-related anxiety, particularly the aftermath of prolonged periods of lockdown. Individuals impacted by anxiety difficulties during the height of the pandemic and now emerging from lockdown are unlikely to return to pre-pandemic levels of normalcy but rather may engage in excessive checking, worrying, and avoidance behaviours as well as threat monitoring directed towards hygiene and infection control. Whilst these behaviours may initially serve to relieve distress, the net result thereof is the likely exacerbation and prolongation of clinical levels of anxious distress [24,25].

The CAS’s focus on the physiological symptoms that manifest from clinical fear and anxiety is a particular strength of this measure. The content of the items—dizziness, sleep disturbance, tonic immobility/motor inhibition, loss of appetite, and abdominal distress—are symptoms that occur across a range of other existing psychological diagnoses involving fear and anxiety (e.g., generalized anxiety disorder, post-traumatic stress disorder, panic attacks). Given the significant inter-item correlations, our results therefore further reinforce this measure’s content validity.

Moreover, the significant and substantially large correlation between the CAS and the FCS indicate that the Arabic version is concurrently valid. The FCS measure has thus far been shown to be associated with psychopathological constructs (i.e., depression, stress, and anxiety) [3,30]. Its positive and significant association with the Arabic CAS therefore further highlights the potential clinical utility of the CAS measure. This point is further reinforced by the finding, in the present study, that symptoms of coronavirus anxiety were more prevalent amongst individuals with a positive COVID-19 diagnosis than those who presented with anxiety symptoms but had not been diagnosed with COVID-19. It therefore appears that individuals who have been infected with the illness have distinct psychological needs that require assessment and treatment, a task that a validated instrument such as the CAS could valuably contribute towards. Some scholars have suggested that a substantial proportion of the world’s population, up to 70%, may be in need of both medical and psychological care in relation to the pandemic [41]. An additional clinical consideration highlighted by our data is the finding that participants with a pre-existing anxiety diagnosis evidenced higher levels of coronavirus anxiety than their peers who evidenced COVID-19 related anxiety but had no premorbid issues with anxiety. This is in concurrence with some of the previous validation studies in Brazil [38] and Turkey [19] and is an especially significant finding as it provides empirical evidence for the contention that individuals with pre-existing mental health difficulties are especially at-risk for developing additional psychological symptoms during the coronavirus pandemic [5]. It further highlights the need for assessment and targeted psychological intervention for this at-risk group of individuals who may fear being infected with COVID-19 and overwhelm emergency and medical care services. Pre-emptive screening and treatment will ensure that this unfavourable scenario does not occur. An additional finding of note is that older individuals presented with higher levels of coronavirus-related anxiety. We did not, on this occasion, assess whether individuals had any pre-existing medical diagnoses that might reliably elevate their risk of an unfavourable outcome if they were to be infected with COVID-19. However, a potential explanation may be that older individuals, compared to younger individuals, may be more likely to have additional medical issues such as respiratory or cardiovascular issues and, as such, may be more anxious about the possibility of becoming infected. Certainly, prior research in the UAE has shown independent associations between both age and the presence of medical diagnoses and increased coronavirus-related anxiety [5]. This association, in turn, appears to impact individuals’ adherence to health-protective measures as fear of infection promotes greater compliance [42].

The clinical utility of a validated measure to identify clinical presentations of anxiety related to COVID-19 may be especially valuable in informing the development and application of psychological interventions to target the maladaptive forms of coping that are typically employed in managing these difficulties. For example, interventions to target worry such as meta-cognitive therapy [43], exposure and response prevention to reduce checking and avoidance behaviours [44], and attention training techniques [43]. The application of these interventions could be applied during both episodes of lockdown and indeed during periods in which restrictions are eased.

### Limitations of the study

Despite this study’s clear utility, the following limitations should be borne in mind. First, while our sample was sufficient to conduct the desired analyses, it is somewhat smaller than the previous CAS validation studies. CFAs are generally large sample analyses. Therefore, future researchers may seek to replicate the design of this study but with a larger sample to ensure that future studies are highly powered. Second, the results of studies relating to outbreaks of disease are highly dependent on timing (i.e., the timepoint within the pandemic at which data is collected). Our data were collected at a time when lockdown procedures had begun to be eased. Government-mandated regulatory procedures were no longer at their most stringent and many public spaces and services had begun to open and restart. Thus, the level of anxiety reported in this study may be an underestimation of the true extent of the issue–had the data been collected two weeks earlier, the estimations of anxiety may, potentially, have been higher. Although, if Nikčević and Spada’s [24] proposition is to be believed, anxiety levels within a society may not necessarily diminish following the easing of lockdown restrictions but rather continue to pervade as life is unlikely to return to pre-lockdown levels of normalcy and individuals are required to adjust to new forms of societal engagement that inevitably include a heightened focus on hygiene and infection control, elements that could prolong anxiety difficulties, particular for those with pre-existing mental health issues. Despite these limitations, our results provide an indication of the extent to which anxiety related to the pandemic has persisted beyond the height of the pandemic in this country. Further longitudinal research will be especially valuable in tracking the progression of fear and anxiety as the pandemic continues to shift and evolve through society.

### Conclusion

The Arabic CAS therefore appears to be psychometrically sound, valid, and reliable and can be used to screen for probable cases of coronavirus-related anxiety amongst Arabic-speaking individuals. This is likely to have a substantial positive contribution to the work of clinicians and mental health researchers in the Arabic-speaking world. However, given the sample size of the present study and the use of a single measure to produce a concurrent validity computation, future studies should endeavour to recruit widely and administer a range of comparable instruments against which the validity of the Arabic CAS could be examined.

## Supporting information

S1 File Complete dataset for the present study (SPSS file). Study dataset.
(SAV)

S2 File Arabic Coronavirus Anxiety Scale (MS Word file). Full CAS survey.
(DOCX)
