# Supplementary material for: Measuring anxiety related to COVID-19: Factor analysis and psychometric properties of the Arabic Coronavirus Anxiety Scale
Source: PLoS One. 2021 Nov 24;16(11):e0260355. doi: 10.1371/journal.pone.0260355 (PMC8612521; doi:10.1371/journal.pone.0260355)
Supplement: S2 File — Full CAS survey. (DOCX) [file pone.0260355.s002.docx]

**Appendix – Arabic Coronavirus Anxiety Scale**

| **كم مرة تعرضت للأحداث التالية على مدى الأسبوعين الماضيين؟** | **أبداً** | **نادراً، أقل من يوم أو يومين** | **عدة أيام** | **أكثر من ٧ أيام** | **تقريباً يومياً على مدى الأسبوعين الماضيين** |
| --- | --- | --- | --- | --- | --- |
| شعرت بالدوار، أو الدوخة، أو الإغماء، عندما قرأت أو سمعت أخباراً عن فيروس كورونا. | ٠ | ١ | ٢ | ٣ | ٤ |
| واجهت صعوبة في النوم أو البقاء نائماً لأني كنت أفكر في فيروس كورونا. | ٠ | ١ | ٢ | ٣ | ٤ |
| شعرت بالعجز عن الحركة أو الجمود عندما فكرت في فيروس كورونا أو تعرضت لمعلومات عنه. | ٠ | ١ | ٢ | ٣ | ٤ |
| فقدت الرغبة في الأكل عندما فكرت في فيروس كورونا أو تعرضت لمعلومات عنه. | ٠ | ١ | ٢ | ٣ | ٤ |
| شعرت بالغثيان أو عانيت من مشاكل في المعدة عندما فكرت في فيروس كورونا أو تعرضت لمعلومات عنه. | ٠ | ١ | ٢ | ٣ | ٤ |

The corresponding English version of the CAS is illustrated below.

| How often have you experienced the following activities over the last 2 weeks? | Not at all | Rare, less than a day or two | Several days | More than 7 days | Nearly every day over the last 2 weeks |
| --- | --- | --- | --- | --- | --- |
| I felt dizzy, lightheaded, or faint, when I read or listened to news about the coronavirus. | 0 | 1 | 2 | 3 | 4 |
| I had trouble falling or staying asleep because I was thinking about the coronavirus. | 0 | 1 | 2 | 3 | 4 |
| I felt paralyzed or frozen when I thought about or was exposed to information about the coronavirus. | 0 | 1 | 2 | 3 | 4 |
| I lost interest in eating when I thought about or was exposed to information about the coronavirus. | 0 | 1 | 2 | 3 | 4 |
| I felt nauseous or had stomach problems when I thought about or was exposed to information about the coronavirus. | 0 | 1 | 2 | 3 | 4 |
